# Supplementary figures and images for: Self-reported decreases in the purchases of selected unhealthy foods resulting from the implementation of warning labels in Mexican youth and adult population
Source: Int J Behav Nutr Phys Act. 2024 Jun 14;21:64. doi: 10.1186/s12966-024-01609-3 (PMC11177525; doi:10.1186/s12966-024-01609-3)

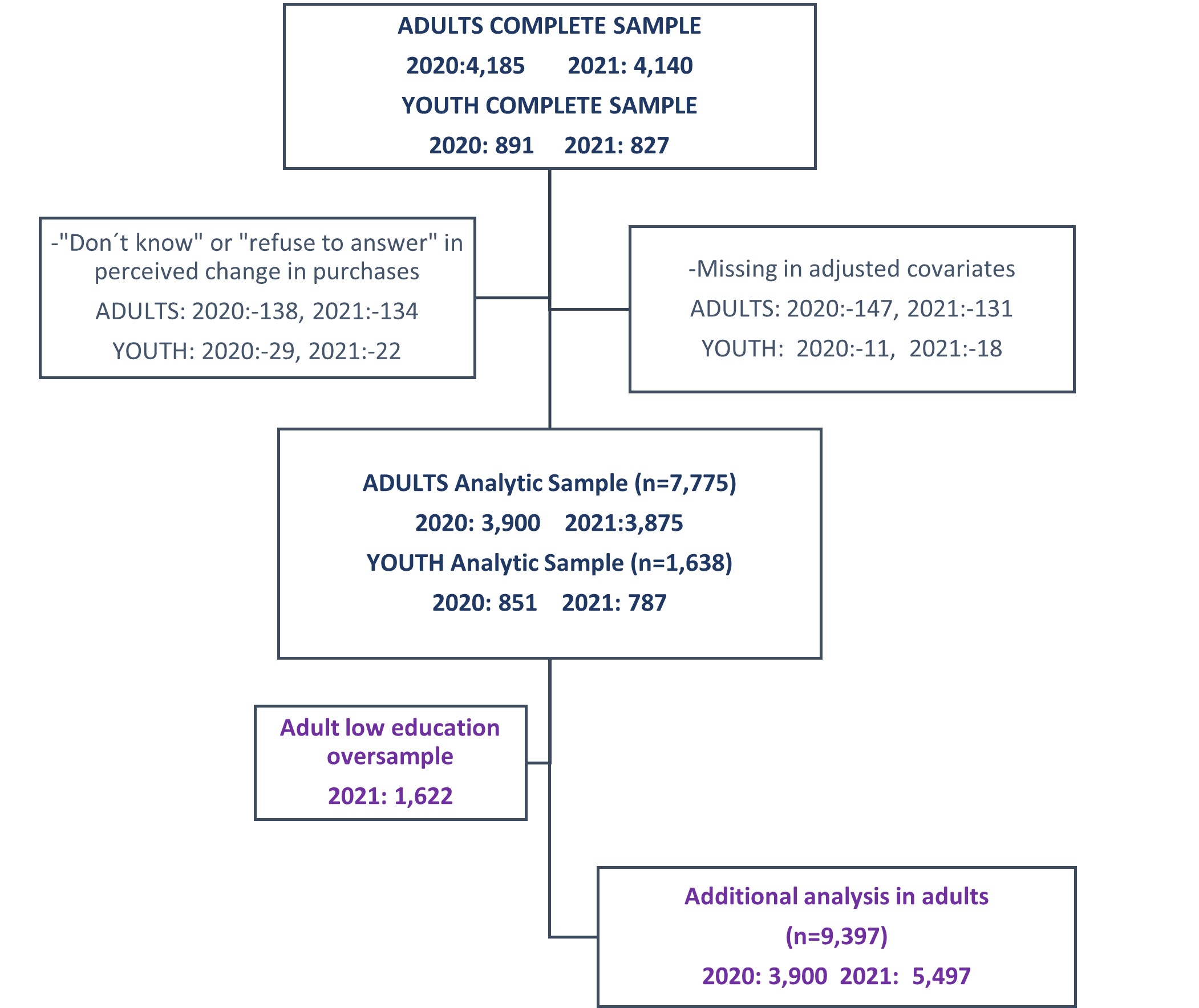

Supplement: Supplementary file 1 — Additional Fig. 1. Participant Flow Diagram with Sample Size for Adults and Youth in the IFPS Study (2019–2021). [file 12966_2024_1609_MOESM1_ESM.jpg]

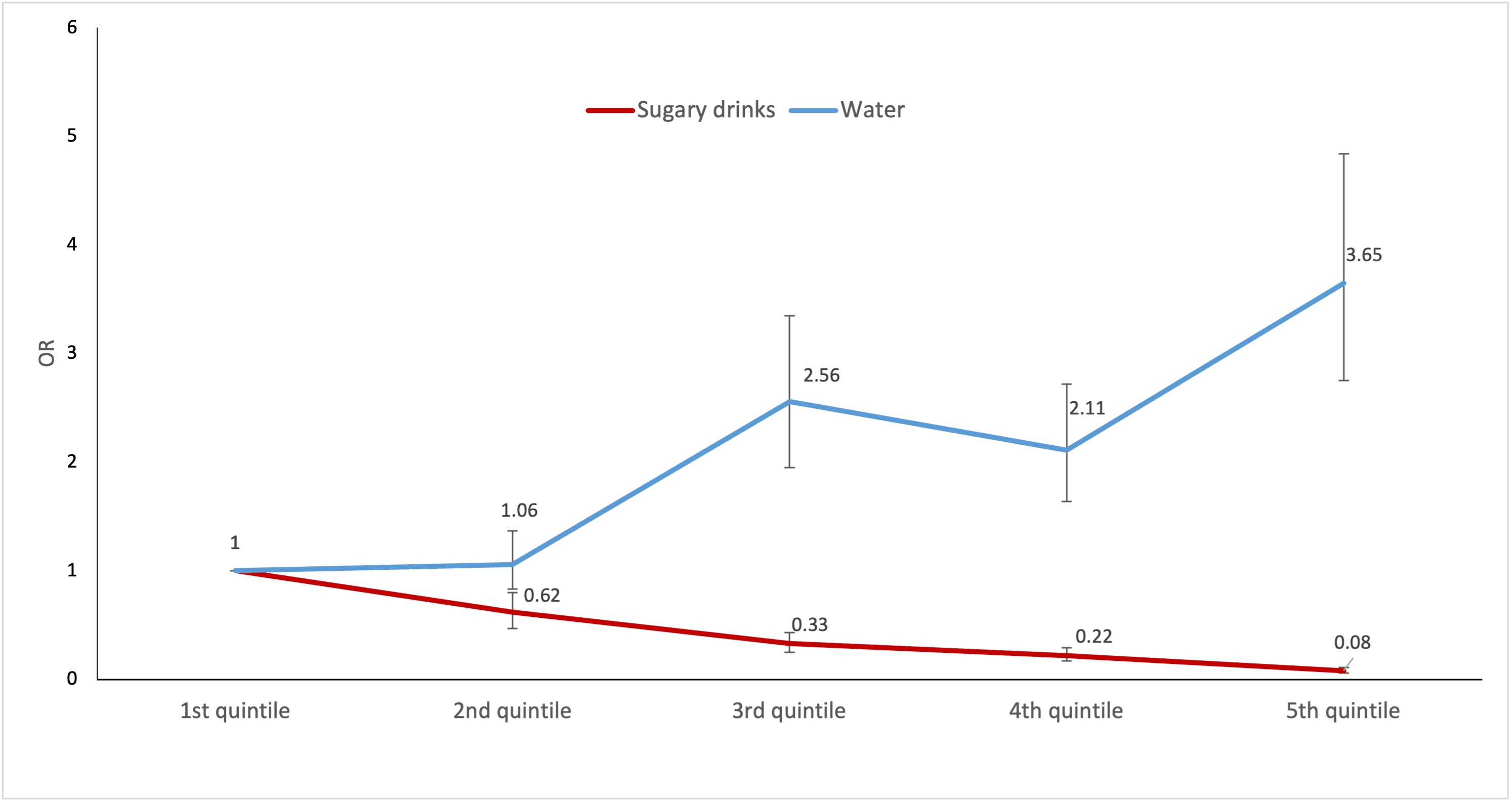

Supplement: Supplementary file 6 — Additional file 4. Odds ratio of perceiving each warning label most useful for choosing healthier foods among Mexican adults and youth, International Food Policy Study, 2020 and 2021. Odds ratios were obtained from logistic regression models. [file 12966_2024_1609_MOESM6_ESM.jpg]
