# Supplementary material for: Self-reported decreases in the purchases of selected unhealthy foods resulting from the implementation of warning labels in Mexican youth and adult population
Source: Int J Behav Nutr Phys Act. 2024 Jun 14;21:64. doi: 10.1186/s12966-024-01609-3 (PMC11177525; doi:10.1186/s12966-024-01609-3)
Supplement: Supplementary file 2 — Additional Fig. 2. Odds ratio of perceiving buying less sugary drinks due to the WLs by quintile of the intake of sugary beverages and water in adults including an oversample with a low education level in 2021, International Food Policy Study, 2020 and 2021 (n = 8,555). Perceived purchase change in sugary drink groups included cola, soda, and sweetened fruit drinks. Sugary drink intake was based on the summed volume of regular soda, sweetened fruit drinks, regular flavored waters or vitamin waters with calories, regular sports drinks, and regular energy drinks. Median intake per quintile: Sugary drinks intake (Q1 = 71 ml, Q2 = 295 ml, Q3 = 526 ml, Q4 = 832 ml, Q5 = 1529 ml), Water intake (Q1 = 0 ml, Q2 = 357 ml, Q3 = 714 ml, Q4 = 1000 ml, Q5 = 1786 ml). Multilevel regression model adjusted by age, sex, indigeneity, educational level, income adequacy, children in the household, nutrition knowledge, role in the food shopping in the household, BMI category and year of the survey. [file 12966_2024_1609_MOESM2_ESM.docx]

| **Additional file 1**. Sociodemographic characteristics of the main sample of adults and oversample with a low-income adequacy. International Food Policy Study 2020 and 2021. | | | | | |
| --- | --- | --- | --- | --- | --- |
|  |  | **2020** | **2021**  **Main sample** | **2021**  **(Including the low-education level oversample)** | **Overall**  **(Including the low-education level oversample.)** |
| n sample |  | 3,900 | 3,875 | 5,497 | 9,397 |
|  |  | **% (95% CI)** | **% (95% CI)** | **% (95% CI)** | **% (95% CI)** |
| Age (years)* |  | 40.5 (39.9, 41.1) | 40.5 (40.3, 41.6) | 40.8 (40.2, 41.5) | 40.7 (40.2, 41.1) |
| Sex | Females | 51.8 (49.9, 53.7) | 52.0 (50.2, 53.8) | 51.9 (49.9, 53.8) | 51.9 (50.5, 53.2) |
|  | Males | 48.2 (46.3, 50.0) | 47.9 (46.1, 49.8) | 48.1 (46.2, 50.0) | 48.1 (46.8, 49.5) |
| Ethnicity | Yes | 19.2 (17.6, 20.9) | 19.2 (17.7, 20.9) | 19.4 (17.9, 20.9) | 19.3 (18.2, 20.4) |
|  | No | 80.8 (79.1, 82.4) | 80.8 (79.1, 82.3) | 80.6 (79.1, 82.1) | 80.7 (79.6, 81.8) |
| Income adequacy | Difficult | 50.2 (48.3, 52.1) | 39.8 (37.9, 41.7) | 47.9 (45.9, 49.8) | 48.8 (47.5, 50.2) |
|  | Neither | 36.1 (34.3, 37.9) | **40.8 (39.0, 42.7)** | 40.8 (38.9, 42.7) | 38.8 (37.5, 40.1) |
|  | Easy | 13.7 (12.6, 15.0) | **19.4 (18.0, 20.8)** | 11.4 (10.3, 12.5) | 12.4 (11.6, 13.2) |
| BMI category^‡^ | Underweight/Normal | 38.0 (36.2, 39.8) | 41.1 (39.3, 43.0) | 32.6 (30.9, 34.4)* | 34.9 (33.6, 36.2) |
|  | Overweight | 31.9 (30.2, 33.8) | 30.6 (28.9, 32.3) | 29.5 (27.7, 31.2) | 30.5 (29.2, 31.8) |
|  | Obesity | 15.3 (13.9, 16.8) | 14.8 (13.5, 16.2) | 17.4 (15.9, 18.9) | 16.5 (15.5, 17.6) |
|  | Missing | 14.7 (13.4, 16.1) | **13.4 (12.1, 14.7)** | 20.5 (18.9, 22.2) | 18.1 (17.0, 19.2) |
| Education level | Low | 21.4 (19.9, 23.0) | 19.1 (17.7, 20.5) | 74.6 (73.0, 75.9)* | 52.3 (50.9, 53.7) |
|  | Medium | 13.6 (12.2, 14.9) | 14.2 (12.9, 15.6) | 10.0 (8.8, 11.4) | 11.5 (10.6, 12.5) |
|  | High | 65.0 (63.2, 66.8) | **66.8 (64.9, 68.5)** | 15.3 (14.5, 16.3) | 36.1 (34.9, 37.4) |
| Children in the household (<18 y old) | No | 51.2 (49.3, 53.1) | 50.4 (48.5, 52.3) | 48.8 (46.9, 50.7) | 49.7 (48.4, 51.1) |
|  | Yes | 48.7 (46.8, 50.6) | 49.6 (47.7, 51.4) | 51.3 (49.4 53.2) | 50.3 (48.9, 51.6) |
| Food shopping role (resume) | Important | 73.6 (72.0, 75.2) | 73.2 (71.6, 74.8) | 68.2 (66.4, 69.9)* | 70.5 (69.2, 71.7) |
|  | Some or None | 26.4 (24.7, 28.0) | 26.7 (25.1, 28.4) | 31.8 (30.0, 33.6) | 29.5 (28.3, 30.8) |
| Nutrition knowledge | Not knowledeable | 31.4 (29.7, 33.1) | 33.9 (32.2, 35.7) | 44.3 (42.4, 46.2)* | 38.9 (37.6, 40.2) |
|  | Somewhat | 55.5 (53.6, 57.4) | 50.8 (48.9, 52.7) | 46.3 (44.3, 48.2) | 50.1 (48.8, 51.5) |
|  | Knowledgeable | 13.1 (11.8, 14.5) | 15.2 (13.9, 16.6) | 9.4 (8.4, 10.6) | 10.9 (10.2, 11.8) |
| The mean and 95% CI are reported for age. **Bold numbers** indicate significant difference between 2020 and main sample in 2021 (p<0.05)  *Indicate significant difference between main sample in 2021 and including low-education level oversample. ^‡^For adults, BMI was categorized as <25 (Underweight or Normal weight), 25-29 (Overweight), and ≥30 (Obesity). For youth, BMI z-score was categorized as ≤1 (Severe thinness/Thinness/Normal), >1 to ≤2 (Overweight), or >2 (Obesity). | | | | | |
