# Supplementary material for: Self-reported decreases in the purchases of selected unhealthy foods resulting from the implementation of warning labels in Mexican youth and adult population
Source: Int J Behav Nutr Phys Act. 2024 Jun 14;21:64. doi: 10.1186/s12966-024-01609-3 (PMC11177525; doi:10.1186/s12966-024-01609-3)
Supplement: Supplementary file 3 — Additional file 1. Adjusted percentage of participants reporting that the warning labels had led them to buy less unhealthy foods overall and across food categories. IFPS 2020 and 2021. The mean and 95% CI are reported for age. Bold numbers indicate significant difference between 2020 and main sample in 2021 (p < 0.05). *Indicate significant difference between main sample in 2021 and including low-education level oversample. ‡For adults, BMI was categorized as < 25 (Underweight or Normal weight), 25–29 (Overweight), and ≥ 30 (Obesity). For youth, BMI z-score was categorized as ≤ 1 (Severe thinness/Thinness/Normal), > 1 to ≤ 2 (Overweight), or > 2 (Obesity). [file 12966_2024_1609_MOESM3_ESM.docx]

| **Additional file 2.** Adjusted percentage of participants reporting that the warning labels had led them to buy less unhealthy foods overall and across food categories. International Food Policy Study, 2020 and 2021. | | | | |
| --- | --- | --- | --- | --- |
|  |  | **Youth** | **Adults** | **Adults** |
|  |  | **(n=1638)** | **(n=7,775)** | **(Including low-education level oversample) (n=9,370)** |
| Food group | **Year** | **% (95% CI)** | **% (95% CI)** | **% (95% CI)** |
| Overall (excluding 100% fruit juice) | 2020 | 39.5 (37.3, 41.7) | 45.4 (44.2, 46.6) | 47.5 (46.2, 48.7) |
|  | 2021 | 37.7 (35.6, 39.8) | 44.2 (43.1, 45.3) | 48.8 (47.7, 49.9) |
|  | Total | 38.6 (37.0, 40.3) | 44.8 (44.0, 45.6) | 48.3 (47.3, 49.1) |
| Cola | 2020 | 51.7 (47.7, 55.8) | 52.7 (50.7, 54.6) | 53.3 (51.1, 55.5) |
|  | 2021 | 47.9 (43.7, 52.0) | 52.1 (50.2, 54.0) | 55.4 (53.6, 57.3) |
|  | Total | 49.9 (47.0, 52.9) | 52.4 (51.0, 53.7) | 54.6 (53.1, 55.9) |
| Soda | 2020 | 51.3 (47.2, 55.3) | 53.0 (51.1, 54.9) | 53.9 (51.8, 56.1) |
|  | 2021 | 47.4 (43.1, 51.6) | 51.3 (49.4, 53.2) | 55.7 (53.8, 57.3) |
|  | Total | 49.4 (46.4, 52.4) | 52.2 (50.8, 53.5) | 54.9 (53.6, 56.3) |
| Diet soda | 2020 | 49.6 (45.5, 53.7) | 50.6 (48.7, 52.5) | 52.6 (50.5, 54.7) |
|  | 2021 | 45.0 (40.8, 49.3) | 48.8 (47.0, 50.7) | 53.5 (51.6, 55.3) |
|  | Total | 47.5 (44.4, 50.4) | 49.7 (48.4, 51.1) | 53.1 (51.7, 54.5) |
| Sweetened fruit drinks | 2020 | 42.5 (38.4, 46.6) | 50.6 (48.7, 52.5) | 51.3 (49.2, 53.4) |
|  | 2021 | 39.3 (35.2, 43.3) | 50.2 (48.3, 52.1) | 52.9 (51.1, 54.8) |
|  | Total | 41.0 (38.1, 43.9) | 50.4 (49.0, 51.7) | 52.2 (50.9, 53.7) |
| Candy or chocolate bars | 2020 | 39.9 (35.9, 43.9) | 46.3 (44.4, 48.3) | 47.6 (45.5, 49.8) |
|  | 2021 | 37.2 (33.2, 41.3) | 45.4 (43.6, 47.3) | **50.9 (49.1, 52.8)** |
|  | Total | 38.6 (35.8, 41.5) | 45.9 (44.5, 47.2) | 49.6 (48.2, 51.0) |
| Snacks such as chips | 2020 | 35.9 (32.0, 39.9) | 45.5 (43.6, 47.4) | 47.3 (45.2, 49.5) |
|  | 2021 | 34.2 (30.2, 38.2) | 42.9 (41.0, 44.7) | 46.9 (45.1, 48.7) |
|  | Total | 35.2 (32.4, 37.9) | 44.2 (42.8, 45.5) | 47.1 (45.7, 48.5) |
| Desserts such as cakes, | 2020 | 38.1 (34.1, 42.1) | 46.3 (44.4, 48.2) | 47.1 (44.9, 49.2) |
| cookies and ice cream | 2021 | 34.6 (30.5, 38.6) | 44.6 (42.7, 46.5) | 48.8 (46.9, 50.7) |
|  | Total | 36.4 (33.6, 39.2) | 45.4 (44.1, 46.8) | 48.1 (46.7, 49.5) |
| Sugary cereals | 2020 | 39.5 (35.5, 43.5) | 48.8 (46.9, 50.8) | 49.2 (47.0, 51.3) |
|  | 2021 | 38.0 (33.9, 42.1) | 49.4 (47.5, 51.2) | 51.9 (50.1, 53.8) |
|  | Total | 38.8 (35.9, 41.6) | 49.1 (47.8, 50.4) | 50.8 (49.4, 52.1) |
| 100% fruit or vegetable juice | 2020 | 21.0 (17.7, 24.4) | 29.6 (27.8, 31.3) | 29.2 (27.3, 31.2) |
|  | 2021 | 18.7 (15.4, 21.9) | **26.7 (25.0, 28.4)** | 27.4 (25.7, 29.1) |
|  | Total | 19.9 (17.6, 22.2) | 28.1 (26.9, 29.3) | 28.2 (26.9, 29.4) |
| Percentages were obtained from logistic regression models for each food group and adjusted by year of the survey, sex, age, indigeneity, income adequacy and BMI category for adults and youth, and additionally adjusted by educational level, children in the household, nutrition knowledge, role in the food shopping in the household for adults. Bold numbers indicate significant difference between 2020 and 2021 (p<0.05) | | | | |
