# Supplementary material for: Self-reported decreases in the purchases of selected unhealthy foods resulting from the implementation of warning labels in Mexican youth and adult population
Source: Int J Behav Nutr Phys Act. 2024 Jun 14;21:64. doi: 10.1186/s12966-024-01609-3 (PMC11177525; doi:10.1186/s12966-024-01609-3)
Supplement: Supplementary file 4 — Additional file 2. Adjusted percentage of self-reported usefulness of each of the warning label in Mexican, youth and adults in 2020 and 2021. Percentages were obtained from logistic regression models for each food group and adjusted by year of the survey, sex, age, indigeneity, income adequacy and BMI category for adults and youth, and additionally adjusted by educational level, children in the household, nutrition knowledge, role in the food shopping in the household for adults. Bold numbers indicate significant difference between 2020 and 2021 (p < 0.05). [file 12966_2024_1609_MOESM4_ESM.docx]

| **Additional file 3.** Adjusted percentage of participants perceiving warning labels as useful in Mexican youth and adults, International Food Policy Study, 2020 and 2021. | | | | |
| --- | --- | --- | --- | --- |
|  |  | **Youth**  **(n=1,638)** | **Adults**  **(n=7,775)** | **Adults**  **(Including lo-education level oversample) (n=9,370)** |
|  | Year | **% (95% CI)** | **% (95% CI)** | **% (95% CI)** |
| Excess Calories | 2020 | 10.3 (7.8, 12.8) | 9.5 (8.4, 10.6) | 8.9 (7.8, 10.1) |
|  | 2021 | 10.8 (8.2, 13.3) | 10.2 (9, 11.5) | 10.6 (9.3, 11.8) |
|  | Total | 10.8 (9.0, 12.6) | 10.3 (9.5, 11.1) | 10.2 (9.3, 11.0) |
| Excess Sodium | 2020 | 3.4 (2.2, 4.7) | 6 (5, 6.9) | 5.5 (4.6, 6.5) |
|  | 2021 | 4.4 (2.4, 6.5) | 6.7 (5.8, 7.7) | 6.3 (5.5, 7.2) |
|  | Total | 4.3 (3.1, 5.4) | 6.6 (5.9, 7.3) | 6.2 (5.6, 6.9) |
| Excess Trans Fat | 2020 | 5.7 (3.8, 7.5) | 5.9 (5, 6.8) | 5.4 (4.4, 6.3) |
|  | 2021 | 5.7 (3.6, 7.9) | 6.3 (5.4, 7.2) | 5.7 (4.9, 6.6) |
|  | Total | 6.0 (4.6, 7.5) | 6.3 (5.7, 7) | 5.8 (5.2, 6.4) |
| Excess Sugars | 2020 | 22.8 (19.4, 26.2) | 16.8 (15.3, 18.2) | 17.8 (16.2, 19.5) |
|  | 2021 | 23.3 (19.9, 26.8) | 18.8 (17.3, 20.2) | 18.1 (16.7, 19.5) |
|  | Total | 23.1 (20.7, 25.6) | 18.2 (17.2, 19.2) | 18.3 (17.3, 19.4) |
| Excess Saturated Fat | 2020 | 8.4 (6.2, 10.6) | 9 (7.9, 10) | 9.2 (7.9, 10.5) |
|  | 2021 | 9.1 (6.6, 11.7) | 10.1 (9, 11.2) | **11.3 (10.2, 12.5)** |
|  | Total | 9.1 (7.3, 10.8) | 9.7 (8.9, 10.5) | 10.6 (9.7, 11.4) |
| None have been useful | 2020 | 15.2 (12.3, 18.2) | 16.2 (14.8, 17.6) | 16.4 (14.8, 18.0) |
|  | 2021 | 14.9 (11.9, 18.1) | 15.4 (14.1, 16.8) | 142. (12.9, 15.5) |
|  | Total | 15.3 (13.2, 17.4) | 16.7 (15.7, 17.7) | 15.8 (14.8, 16.8) |
| All have been equally useful | 2020 | 32.4 (28.5, 36.4) | 34.1 (32.2, 35.9) | 34.6 (32.5, 36.6) |
|  | 2021 | 29.6 (25.9, 33.4) | **29.6 (27.9, 31.3)** | **31.4 (29.7, 33.2)** |
|  | Total | 31.3 (28.6, 33.9) | 32.1 (30.9, 33.4) | 33.0 (31.7, 34.3) |
| Percentages were obtained from logistic regression models for each WLs and adjusted by year of the survey, sex, age, indigeneity, income adequacy and BMI category for youth and adults, and additionally adjusted by education level, children in the household, nutrition knowledge, and food shopping role in the household for adults.  **Bold numbers** indicate significant difference between 2020 and 2021 (p<0.05) | | | | |
