# Supplementary material for: Self-reported decreases in the purchases of selected unhealthy foods resulting from the implementation of warning labels in Mexican youth and adult population
Source: Int J Behav Nutr Phys Act. 2024 Jun 14;21:64. doi: 10.1186/s12966-024-01609-3 (PMC11177525; doi:10.1186/s12966-024-01609-3)
Supplement: Supplementary file 5 — Additional file 3. Odds ratio of perceiving each warning label most useful for food decision making in Mexican adults and youth in 2020 and 2021. Percentages were obtained from logistic regression models for each WLs and adjusted by year of the survey, sex, age, indigeneity, income adequacy and BMI category for youth and adults, and additionally adjusted by education level, children in the household, nutrition knowledge, and food shopping role in the household for adults. Bold numbers indicate significant difference between 2020 and 2021 (p < 0.05). [file 12966_2024_1609_MOESM5_ESM.docx]

| Additional file 4. Odds ratio of perceiving each warning label most useful for choosing healthier foods among Mexican adults and youth, International Food Policy Study, 2020 and 2021. | | | | | | | | |
| --- | --- | --- | --- | --- | --- | --- | --- | --- |
| **Adults (n=7,752)** | WL | Calories | Sodium | Trans Fat | Sugars | Saturated fat | None | All |
| Category | Subpopulation | OR (95% CI) | OR (95% CI) | OR (95% CI) | OR (95% CI) | OR (95% CI) | OR (95% CI) | OR (95% CI) |
| Age | Years | **0.98 (0.98, 0.99)** | 1 (0.99, 1.01) | 1 (0.99, 1) | **0.99 (0.98, 0.99)** | 1 (0.99, 1) | **1.01 (1, 1.01)** | **1.01 (1.01, 1.02)** |
| Sex | Female | 1 | 1 | 1 | 1 | 1 | 1 | 1 |
|  | Male | 0.95 (0.79, 1.14) | **0.76 (0.61, 0.94)** | 0.79 (0.62, 1) | **1.28 (1.11, 1.48)** | 0.98 (0.82, 1.17) | **1.19 (1.03, 1.38)** | 0.9 (0.8, 1.01) |
| Indigeneity | No | 1 | 1 | 1 | 1 | 1 | 1 | 1 |
|  | Yes | **1.4 (1.11, 1.76)** | 0.97 (0.71, 1.34) | 1.2 (0.87, 1.65) | **1.23 (1.03, 1.47)** | 1.12 (0.87, 1.44) | **0.56 (0.45, 0.7)** | 0.93 (0.79, 1.1) |
| Educational level | High | 1 | 1 | 1 | 1 | 1 | 1 | 1 |
|  | Medium | **0.71 (0.54, 0.94)** | 0.95 (0.67, 1.34) | 1.14 (0.82, 1.58) | 0.94 (0.76, 1.17) | 0.91 (0.68, 1.21) | 0.98 (0.79, 1.22) | **1.22 (1.02, 1.45)** |
|  | Low | 0.84 (0.66, 1.06) | 0.78 (0.59, 1.02) | 0.75 (0.55, 1.03) | 1.11 (0.93, 1.32) | 1.11 (0.89, 1.4) | 0.98 (0.81, 1.18) | 1.12 (0.96, 1.3) |
| Income adequacy | Difficult | **0.69 (0.54, 0.88)** | 1.35 (0.99, 1.86) | 1.13 (0.83, 1.55) | 0.91 (0.75, 1.1) | 0.85 (0.66, 1.09) | 1.16 (0.93, 1.44) | 1.13 (0.95, 1.34) |
|  | Neither | **0.76 (0.6, 0.96)** | **1.48 (1.09, 2.01)** | 1.07 (0.79, 1.45) | 0.9 (0.74, 1.08) | 0.85 (0.67, 1.08) | 1.03 (0.82, 1.28) | 1.16 (0.98, 1.38) |
|  | Easy | 1 | 1 | 1 | 1 | 1 | 1 | 1 |
| Children in the household | No | 1 | 1 | 1 | 1 | 1 | 1 | 1 |
|  | Yes | 1.06 (0.88, 1.28) | **1.33 (1.05, 1.67)** | 1.04 (0.83, 1.31) | **1.32 (1.15, 1.53)** | 1.1 (0.92, 1.33) | **0.76 (0.66, 0.88)** | **0.84 (0.75, 0.95)** |
| Nutritional knowledge | Little | 1 | 1 | 1 | 1 | 1 | 1 | 1 |
|  | Somewhat know | 1.07 (0.88, 1.3) | 0.89 (0.7, 1.13) | 1.29 (0.99, 1.69) | 1.01 (0.87, 1.17) | 1.14 (0.93, 1.4) | **0.57 (0.49, 0.67)** | **1.28 (1.12, 1.46)** |
|  | Knowledgeable | 1.21 (0.92, 1.58) | 1.16 (0.81, 1.67) | 1.31 (0.9, 1.91) | 1.05 (0.84, 1.31) | 0.87 (0.63, 1.2) | **0.51 (0.4, 0.65)** | **1.29 (1.06, 1.56)** |
| Shopping role | No | 1 | 1 | 1 | 1 | 1 | 1 | 1 |
|  | Yes | 0.87 (0.71, 1.07) | 1.01 (0.79, 1.29) | 1.03 (0.76, 1.41) | 1.14 (0.96, 1.35) | 1.13 (0.92, 1.39) | 0.97 (0.83, 1.14) | 0.93 (0.81, 1.06) |
| BMI category | Normal | 1 | 1 | 1 | 1 | 1 | 1 | 1 |
|  | Overweight | 1.2 (0.96, 1.49) | 0.91 (0.7, 1.18) | 1.14 (0.87, 1.49) | 0.95 (0.8, 1.13) | 0.84 (0.68, 1.04) | 1.14 (0.95, 1.35) | 0.95 (0.82, 1.09) |
|  | Obesity | 1.16 (0.88, 1.53) | 1.08 (0.78, 1.49) | **0.66 (0.45, 0.97)** | 1.09 (0.88, 1.35) | **0.71 (0.53, 0.96)** | **1.25 (1.02, 1.55)** | 0.94 (0.78, 1.13) |
|  | Missing | 1.23 (0.95, 1.6) | **0.61 (0.42, 0.87)** | 1.23 (0.88, 1.73) | 1.17 (0.95, 1.45) | 0.91 (0.68, 1.2) | 1 (0.79, 1.26) | 0.9 (0.74, 1.09) |
| Year | 2020 | 1 | 1 | 1 | 1 | 1 | 1 | 1 |
|  | 2021 | 1.08 (0.91, 1.3) | 1.14 (0.91, 1.42) | 1.07 (0.85, 1.34) | 1.15 (1, 1.32) | 1.14 (0.96, 1.37) | 0.95 (0.82, 1.09) | **0.81 (0.72, 0.92)** |
|  |  |  |  |  |  |  |  |  |
| **Adults** (main+oversample)  **(n=9,370)** | WL | Calories | Sodium | Trans Fat | Sugars | Saturated fat | None | All |
| Category | Subpopulation | OR (95% CI) | OR (95% CI) | OR (95% CI) | OR (95% CI) | OR (95% CI) | OR (95% CI) | OR (95% CI) |
| Age | Years | **0.99 (0.98, 1)** | 1 (0.99, 1.01) | 1 (0.99, 1.01) | **1.0 (0.99, 1)** | **1 (0.99, 1)** | **1.01 (1.01, 1.02)** | **1.02 (1.01, 1.02)** |
| Sex | Female | 1 | 1 | 1 | 1 | 1 | 1 | 1 |
|  | Male | 0.96 (0.8, 1.16) | **0.79 (0.63, 0.99)** | 1 (0.79, 1.27) | **1.33 (1.15, 1.54)** | 1.02 (0.85, 1.22) | 1.14 (0.98, 1.33) | **0.83 (0.74, 0.94)** |
| Indigeneity | No | 1 | 1 | 1 | 1 | 1 | 1 | 1 |
|  | Yes | **1.33 (1.06, 1.65)** | 1.02 (0.76, 1.38) | 1.23 (0.91, 1.65) | 1.09 (0.92, 1.31) | 1.08 (0.85, 1.36) | **0.65 (0.52, 0.8)** | 0.98 (0.83, 1.14) |
| Educational level | High | 1 | 1 | 1 | 1 | 1 | 1 | 1 |
|  | Medium | 0.95 (0.69, 1.32) | 0.92 (0.62, 1.35) | 1.1 (0.76, 1.61) | 0.82 (0.64, 1.04) | 1.13 (0.84, 1.52) | 0.89 (0.7, 1.13) | 1.18 (0.97, 1.44) |
|  | Low | **0.82 (0.68, 1)** | **0.79 (0.61, 1.01)** | 0.79 (0.6, 1.04) | **1.2 (1.02, 1.4)** | 1.16 (0.95, 1.42) | **0.83 (0.7, 0.99)** | 1.15 (1.01, 1.32) |
| Income adequacy | Easy | 1 | 1 | 1 | 1 | 1 | 1 | 1 |
|  | Neither | 0.89 (0.69, 1.16) | 1.33 (0.94, 1.88) | 1.00 (0.70, 1.41) | 0.94 (0.76, 1.16) | 0.84 (0.65, 1.10) | 0.97 (0.76, 1.24) | 1.12 (0.92, 1.34) |
|  | Difficult | 0.77 (0.59, 1.00) | 1.37 (0.96, 1.93) | 1.22 (0.86, 1.72) | 0.94 (0.77, 1.15) | 0.89 (0.68, 1.16) | 1.04 (0.81, 1.32) | 1.05 (0.87, 1.26) |
| Children in the household | No | 1 | 1 | 1 | 1 | 1 | 1 | 1 |
|  | Yes | 0.98 (0.81, 1.18) | **1.33 (1.05, 1.67)** | 1.2 (0.95, 1.53) | **1.27 (1.1, 1.47)** | 1.05 (0.87, 1.26) | 0.89 (0.76, 1.04) | **0.82 (0.73, 0.93)** |
| Nutritional knowledge | Little | 1 | 1 | 1 | 1 | 1 | 1 | 1 |
|  | Somewhat know | 1.19 (0.97, 1.46) | 0.81 (0.64, 1.04) | **1.45 (1.1, 1.9)** | 0.96 (0.82, 1.11) | 1.16 (0.95, 1.42) | **0.58 (0.49, 0.68)** | **1.24 (1.09, 1.41)** |
|  | Knowledgeable | 1.18 (0.87, 1.59) | 1.01 (0.68, 1.48) | 1.32 (0.88, 1.96) | 1.07 (0.83, 1.36) | 1.01 (0.73, 1.41) | **0.49 (0.38, 0.63)** | **1.29 (1.05, 1.59)** |
| Shopping role | Some or none | 1 | 1 | 1 | 1 | 1 | 1 | 1 |
|  | Most | 1.07 (0.87, 1.33) | 0.99 (0.77, 1.29) | 1.07 (0.81, 1.42) | 1.09 (0.92, 1.3) | 1.15 (0.94, 1.42) | 0.89 (0.75, 1.06) | 0.93 (0.81, 1.06) |
| BMI category | Normal | 1 | 1 | 1 | 1 | 1 | 1 | 1 |
|  | Overweight | 1.06 (0.84, 1.35) | 0.96 (0.73, 1.26) | 1.11 (0.84, 1.48) | 1 (0.84, 1.2) | 1.03 (0.83, 1.28) | 1 (0.83, 1.2) | 0.97 (0.83, 1.12) |
|  | Obesity | 1.22 (0.92, 1.62) | 0.98 (0.7, 1.36) | 0.68 (0.47, 1.01) | 1.15 (0.93, 1.43) | 0.89 (0.67, 1.19) | 1.2 (0.96, 1.49) | 0.87 (0.73, 1.05) |
|  | Missing | 1.22 (0.94, 1.59) | **0.61 (0.43, 0.87)** | 1.05 (0.74, 1.5) | 1.05 (0.85, 1.29) | 1.05 (0.81, 1.36) | 1.21 (0.97, 1.52) | 0.88 (0.73, 1.05) |
| Year | 2020 | 1 | 1 | 1 | 1 | 1 | 1 | 1 |
|  | 2021 | 1.21 (1, 1.45) | 1.15 (0.92, 1.45) | 1.08 (0.85, 1.38) | 1.02 (0.88, 1.19) | **1.26 (1.04, 1.52)** | **0.85 (0.73, 0.99)** | **0.87 (0.77, 0.99)** |
| Youth (n=1,671) | WL | Calories | Sodium | Trans Fat | Sugars | Saturated fat | None | All |
| Category | Subpopulation | OR (95% CI) | OR (95% CI) | OR (95% CI) | OR (95% CI) | OR (95% CI) | OR (95% CI) | OR (95% CI) |
| Age | Years | 0.94 (0.79, 1.13) | 0.98 (0.77, 1.23) | 1.04 (0.83, 1.29) | 0.98 (0.87, 1.11) | 1.01 (0.83, 1.22) | 1.09 (0.94, 1.26) | 1 (0.89, 1.12) |
| Sex | Female | 1 | 1 | 1 | 1 | 1 | 1 | 1 |
|  | Male | 0.77 (0.52, 1.13) | 1.12 (0.62, 1.99) | 0.82 (0.49, 1.37) | 1.06 (0.8, 1.4) | 1.01 (0.67, 1.54) | 0.98 (0.7, 1.36) | 1.14 (0.89, 1.47) |
| Indigeneity | No | 1 | 1 | 1 | 1 | 1 | 1 | 1 |
|  | Yes | 1.03 (0.59, 1.79) | 0.51 (0.16, 1.61) | 1.75 (0.91, 3.39) | 1.21 (0.81, 1.79) | 1.55 (0.92, 2.6) | 0.81 (0.48, 1.37) | 0.76 (0.52, 1.1) |
| Income adequacy | Easy | 1 | 1 | 1 | 1 | 1 | 1 | 1 |
|  | Neither | 0.65 (0.41, 1.03) | 0.78 (0.41, 1.49) | 1.23 (0.66, 2.28) | 1.06 (0.73, 1.54) | 0.84 (0.49, 1.43) | 1.19 (0.74, 1.91) | 1.14 (0.82, 1.58) |
|  | Difficult | **0.59 (0.37, 0.96)** | **0.46 (0.22, 0.97)** | 0.74 (0.36, 1.52) | 0.90 (0.61, 1.34) | 0.96 (0.54, 1.70) | 1.52 (0.94, 2.45) | 1.37 (0.98, 1.90) |
| BMI category | Normal | 1 | 1 | 1 | 1 | 1 | 1 | 1 |
|  | Overweight | 1.34 (0.83, 2.16) | 0.84 (0.43, 1.65) | 1.59 (0.83, 3.07) | 0.93 (0.66, 1.31) | 0.66 (0.36, 1.2) | 1.2 (0.78, 1.84) | 0.87 (0.63, 1.19) |
|  | Obesity | 1.18 (0.52, 2.7) | 1.15 (0.33, 4.03) | 0.84 (0.24, 2.95) | 0.75 (0.38, 1.52) | 1.29 (0.54, 3.06) | 1.21 (0.6, 2.42) | 0.94 (0.53, 1.66) |
|  | Missing | 0.96 (0.58, 1.57) | 0.84 (0.4, 1.73) | 1.15 (0.59, 2.25) | 1.03 (0.71, 1.47) | 1.26 (0.73, 2.16) | 1.07 (0.69, 1.67) | 0.88 (0.63, 1.24) |
| Year | 2020 | 1 | 1 | 1 | 1 | 1 | 1 | 1 |
|  | 2021 | 1.06 (0.73, 1.52) | 1.31 (0.77, 2.22) | 1.02 (0.61, 1.72) | 1.04 (0.79, 1.37) | 1.1 (0.72, 1.7) | 0.98 (0.71, 1.36) | 0.88 (0.69, 1.13) |
| Odds ratios were obtained from logistic regression models. | | | | | | | | |
